# Long-Term Stability in Electronic Properties of Textile Organic Electrochemical Transistors for Integrated Applications

**DOI:** 10.3390/ma16051861

**Published:** 2023-02-24

**Authors:** Riccardo Manfredi, Filippo Vurro, Michela Janni, Manuele Bettelli, Francesco Gentile, Andrea Zappettini, Nicola Coppedè

**Affiliations:** 1 IMEM-CNR Institute of Materials for Electronics and Magnetism, Italian National Research Council, Parco Area delle Scienze, 37/A, 43124 Parma, Italy; 2 Nanotechnology Research Center, Department of Experimental and Clinical Medicine, University of Magna Graecia, 88100 Catanzaro, Italy

**Keywords:** conductive polymer, organic electrochemical transistor, ageing, biosensor, thin film

## Abstract

Organic electrochemical transistors (OECTs) have demonstrated themselves to be an efficient interface between living environments and electronic devices in bioelectronic applications. The peculiar properties of conductive polymers allow new performances that overcome the limits of conventional inorganic biosensors, exploiting the high biocompatibility coupled to the ionic interaction. Moreover, the combination with biocompatible and flexible substrates, such as textile fibers, improves the interaction with living cells and allows specific new applications in the biological environment, including real-time analysis of plants’ sap or human sweat monitoring. In these applications, a crucial issue is the lifetime of the sensor device. The durability, long-term stability, and sensitivity of OECTs were studied for two different textile functionalized fiber preparation processes: (i) adding ethylene glycol to the polymer solution, and (ii) using sulfuric acid as a post-treatment. Performance degradation was studied by analyzing the main electronic parameters of a significant number of sensors for a period of 30 days. RGB optical analysis were performed before and after the treatment of the devices. This study shows that device degradation occurs at voltages higher than 0.5 V. The sensors obtained with the sulfuric acid approach exhibit the most stable performances over time.

## 1. Introduction

Organic electrochemical transistors (OECTs) are extensively used in fields, such as bioengineering, bioelectronics, and biomedical nanotechnology, as biosensors, bio-devices, and constituents of the bio-interface between inorganic materials and living matters [1,2]. Since OECTs generate electrical signals, they can be easily integrated into larger systems and acquire information selectively, accurately, ubiquitously, and with astonishingly low latency time. Because of these properties, OECTs have found application in Internet of Things (IoT) sensor networks, allowing the collection and exchange of data *continuously* without human intervention [3,4,5,6,7]. However, in these and other similar applications, sensors have to be bio-inert, bio-compatible, and capable of operating for a long time in biological fluids [8] to ensure correct functioning.

OECTs made of the conducting polymer Poly (3,4-ethylenedioxythiophene) (PEDOT) doped with polystyrene sulfonate (PSS) can meet these requirements [7]. PEDOT: PSS is one of the most widely used industrial polymers due to its high conductivity, stability, and ease of use [9]. OECTs based on PEDOT:PSS can convert the concentration of ions in a solution into an electric signal efficiently, reversibly, and with a time constant of a few seconds to a few minutes, enabling an accurate analysis of complex mixtures and biological fluids [1,10,11,12].

To optimize performance and improve efficiency even further, scientists have recently developed a sensing device that combines the electrical properties of PEDOT:PSS with the mechanical and biomimetic characteristics of textiles [13]. While the design of this sensing device is relatively simple, i.e., a cotton thread functionalized with PEDOT:PSS, due to the characteristics of natural fibers, such OECT devices exhibit high flexibility, high stability, and the ability to efficiently adsorb electrolyte solutions, enabling the efficient analysis of analytes in solutions [2,13,14].

Because of these characteristics, textile-based OECT devices have been used in a variety of applications, including biological, healthcare, and agricultural applications [11,12,15].

In references [4,10], OECTs integrated into a T-shirt were used to monitor the health status of athletes while training. Placed in contact with the skin, the device enabled real-time monitoring of human sweat, specifically the concentration of mineral salts dispersed in sweat, and assessment of the physiological conditions of athletes over time [10,13,16,17].

In another application, a biomimetic textile OECT was inserted within a plant’s stem, allowing the measurement of the plant’s sap concentration and saturation [6]. Different from all other reported methods for plant sensing, this OECT device allows the measurement of changes occurring in vivo in the plant’s sap during the entire plant growth cycle non-destructively, non-invasively, and without altering plant physiology. The device (Bioristor) was successfully tested in plants subjected to drought and salt stress and different environmental conditions, such as vapor pressure deficit [12,18,19].

In analyzing the performance of OECT devices, commonly adopted metrics are selectivity, sensibility, and durability. Durability is particularly relevant in agriculture as experiments can last up to the plants’ entire life cycle of operation.

Previous studies have illustrated possible strategies to improve the conductivity of PEDOT:PSS [20,21,22], i.e., by (i) adding ethylene glycol—a plasticizer—as an external agent or (ii) applying a post-treatment with sulfuric acid. However, none of these studies have examined whether similar treatments can either improve or hamper the device’s material strength and longevity.

In this work, in acknowledgement of the fact that no reported studies have focused on the mechanical stability of textile/PEDOT:PSS OECTs following chemical process and treatments, we examined how the long-term stability of textile OECTs is influenced by the aforementioned treatments. In our experiments in which the operating voltage at the gate and at the channel was varied over large intervals, we found the optimal operating conditions that assured maximum life of the device. For voltages lower than 0.5 V, textile/PEDOT:PSS OECTs worked correctly without interruption up to 30 days.

## 2. Experimental Section

### 2.1. Sensor Design

OECTs were fabricated using a polypropylene fiber functionalized with conductive PEDOT:PSS polymer [13]. The conductive thread was used as the sensor’s channel and the sensor’s gate. The polypropylene fiber is a white multi-fiber thread that can absorb by capillarity the PEDOT:PSS and the aqueous sample—similarly to a cotton fiber [13,23]. PEDOT:PSS is a p-conductive polymer largely used for its high stability and its ability to interact with ions in solutions [14]. The sensor has three electrodes: (i) the drain, (ii) the source positioned at the channel’s ends, and (iii) the gate, which is separated by an electrolytical solution from the channel. The voltage applied between the drain and the source, V_ds_, generates the current I_ds_, while the voltage applied between the gate and the source, V_gs_, generates the current at the gate I_gs_. I_gs_ results from cations moving from the electrolyte to the channel. PEDOT:PSS in the channel reacts with these ions, and the thread conductivity decreases, as described by the following de-doping equation at equilibrium [24]:

(1) $${\mathrm{PEDOT}}^{+}:{\mathrm{PSS}}^{-}+M^{+}+e^{-}=\mathrm{PEDOT}+M^{+}:{\mathrm{PSS}}^{-}$$

where M^+^ is a metal cation in the solution; e^−^ is an electron from the channel; and PEDOT^+^:PSS^−^ is the PEDOT, which is hole-doped and stabilized with the counter-anion PSS. The conductive threads in the OECT were prepared following two different protocols: (a) by adding ethylene glycol in the solution, and (b) by applying a sulfuric acid post-treatment [20,25,26].

### 2.2. Functionalization with Ethylene Glycol in Solution

The functionalization was accomplished by soaking the threads in 100 mL of aqueous solution of PEDOT:PSS (Clevious PH1000) with 10% ethylene glycol and 2% DBSA surfactant. Then, the solution was stirred for 10 min. The polypropylene (PP) threads were previously cleaned under oxygen plasma. Functionalization was performed repeatedly for three times. The threads were then heated at 120 °C for 15 min.

### 2.3. Functionalization with Sulfuric Acid Post-Treatment

In this case, functionalization was accomplished by soaking the threads in an aqueous solution of 100 mL of PEDOT:PSS (Clevious PH1000) with 2% of DBSA. The PP threads were previously cleaned under oxygen plasma. After three cycles of functionalization and sample post-treatment on a hot plate at 120 °C for 15 min, the threads were treated with a solution of sulfuric acid (H_2_SO_4_) and DI water at 95% *v*/*v* for 20 min. The samples were then washed with DI water to remove the excess of sulfuric acid and heated at 120 °C for 15 min. This process was repeated two times.

### 2.4. Device Fabrication and Characterization

**Figure 1:**
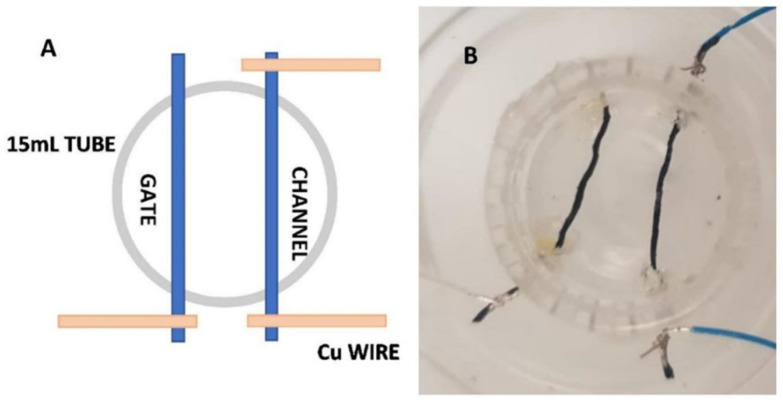
Sketch of the OECT geometry used in all the experiments (**A**) and prototype of a real OECT device (**B**).

Upon functionalization, the threads were connected to copper wires by silver paste. The drain and source contacts—fixed at the end of one thread—formed the channel; a second thread was used as the gate electrode. The threads were then inserted in a 15 mL plastic test tube, as shown in Figure 1. In this configuration, the length of the threads was 15 mm, with a thread-to-thread distance of 7 mm. The experiments were performed by filling the test tube with a solution of NaCl at a concentration of 5 mM, which is characteristic of a plant’s sap. The electric response of the sensor was examined by stimulating the device with a continuous cycle of on/off gate voltages for the whole duration of the experiment.

### 2.5. Study Design

In a first test campaign, 13 sensors were prepared with the first method (functionalization with ethylene glycol) and 10 sensors were prepared with the second method (functionalization with sulfuric acid). The sensors were tested for 34 days with cycles of 18 min (gate off) and 6 min (gate on), applying a voltage value of 0.1 V at the channel and of 1 V at the gate. We carried out the experiments using different NaCl concentrations with the aim of calibrating the sensor, i.e., 0.1 mM, 1 mM, 5 mM, and 10 mM.

In a second test campaign, we varied the voltage at the gate using values of 1 V, 0.75 V, and 0.5 V, respectively. In these tests, we switched the voltage at the gate cyclically between an on/ and an off/ state every 6 min. The sensors were prepared using the treatments described in Section 2.2 and Section 2.3 to examine how the gate’s lifetime varied as a function of the applied gate voltage for different sample treatments. For each combination of the parameters, the experiments were repeated using three different sensors (3 sample repeats).

We used different metrics to assess the performance of the sensors:

(i) The *channel current* at the beginning of each cycle (I_0_), which depends on the hole mobility of the PEDOT:PSS channel [19].
(ii) The *sensor response* (R), calculated as the relative variation of the current at the channel measured for the on/off V_g_ cycle: (I − I_0_)/I_0_. In the existing literature regarding organic electrochemical sensors’ (OECTs) response, modulation and sensitivity are synonym and are often used interchangeably. Thus, in the following sections of this paper, the sensor *response* is occasionally called *modulation* or *sensitivity*.
(iii) The *gate–source current* (I_gs_), measured as the maximum current flowing from the gate to the source when V_g_ is applied.
(iv) The *sensitivity* (S), defined as the slope of the response R against the concentration of the solute, as measured for a constant V_g_.
(v) The *time constant* *τ*, i.e., the time required for the system’s response $f\left(t\right)$ to reach the 66% of its steady-state value [27], which is found by non-linear fitting of data with an exponential curve of the form $f\left(t\right)=A\left(1-e^{t/\tau }\right)$, where A is a model parameter and t is the time. *τ* is related to the reaction kinetics of de-doping [28]: it depends on the diffusion of cations into the channel and represents an important parameter to measure the effectiveness of the channel de-doping rate.

### 2.6. Image Analysis of PEDOT:PSS Functionalized Cotton Fibers

We used RGB (Red, Green, and Blue) analysis to examine the conductive fibers before and after the tests. RGB analysis is a color-based technique used in a variety of different applications, such as sample classification and segmentation [29,30]. It is a point-to-point representation of the thread in a color space—expressed as a combination of Red (R), Green (G), and Blue (B) values—that can vary from 0 (lighter tones) to 255 (darker tones). The grayscale value transform (Y) then converts the measured values of R, G, and B into one single parameter—the intensity. It is calculated as the weighted average of R, G, and B as follows: *Y* = 0.299(*Red*) + 0.587(*Green*) + 0.114(*Blue*). This transformation was defined by the National Television Standards Committee (NTSC) [29], and it is now the consolidated method to calculate grayscale values. In this study, we further normalized the values of Y between 0 (corresponding to a completely black point) to 1 (corresponding to a completely white point). The grayscale values were obtained by using Image-J. The thread images, with 300 dpi resolution, were taken with a photo camera, CANON-EOS 6D. The analysis was performed for each tested sample treatment. One image was taken for each different fiber topology.

## 3. Results

In this section, we illustrate how the OECT sensor device performs at a constant gate voltage of 1V following two different sample preparations: (i) addition of ethylene glycol (EG) to the PEDOT:PSS (EG samples), and (ii) post-treatment of the PEDOT:PSS channel with H_2_SO_4_ (sulfuric acid, SA samples).

### 3.1. Exploitation of EG as the Plasticizer

**Figure 2:**
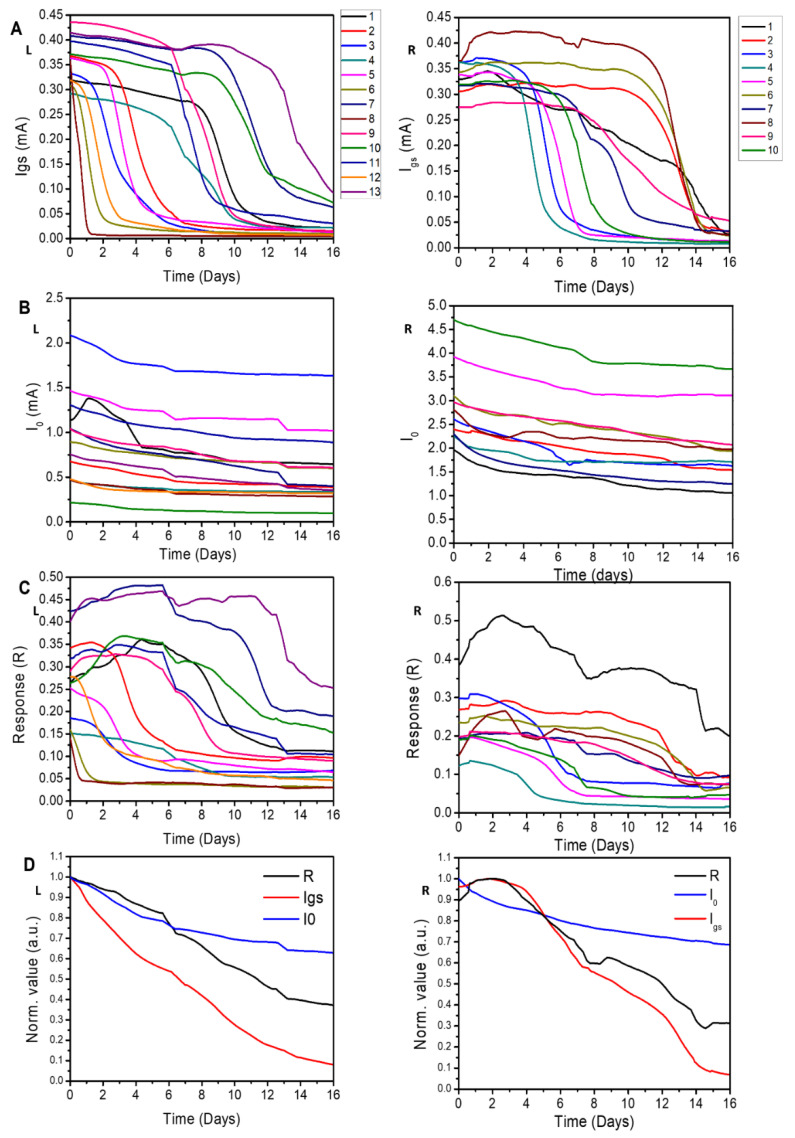
The performance of 13 devices using EG as the plasticizer in the PEDOT:PSS formulation (L) and the performance of 10 devices using the acid sulfuric post-treatment (R). The performance indices in time, by applying V_g_ = 1 V and V_ds_ = −0.1 V for 16 days with 5 mM of NaCl solution, are the gate–source current (I_gs_) (**A**), source–drain current (I_0_) (**B**), response (R) (**C**), and normalized average value of R, I_gs_, and I_0_ (**D**).

The diagrams in Figure 2 show the variation over time in I_gs_ (Figure 2A, L), I_0_ (Figure 2B, L), and R (Figure 2C, L) measured in 13 devices. The devices show similar behavior. The gate–source current I_gs_ (Figure 2A, L) starts from values comprised between 0.27 and 0.45 mA at the beginning of the experiment to a value falling in the 0–0.12 mA range after 16 days. This transition may be divided into three phases: (i) a fairly constant value is maintained for a time that varies between 1 and approximately 10 days, depending on the sensor device; (ii) a steep linear reduction in the current occurring in approximately one to three days; and (iii) a settling phase in which the current of the sensor device plunges to zero. Figure 2B, L illustrates that the value of I_0_ decreases with time. For all the tested devices, the drain–source current is quasi-stable, with an overall reduction in intensity below 30% of the initial value. The response R of the OECT for all 13 devices is reported in Figure 2C, L. The higher the response, the higher the performance of the device. For two of the tested devices, the response falls sharply to about 0.5 in approximately one day. For the remaining sensor devices, R is constant for a time that varies between 2 and 14 days; then, it smoothly transitions to a lower steady-state value, comprised between 0.01 and 0.3, denoting a moderate-to-low efficiency of the sensor devices.

To compare the results and assess the performance of the devices quantitatively, we report in Figure 2D, L the *normalized* values of the gate–source current (I_gs_), drain–source current (I_0_), and response (R), which were measured over time and averaged over 13 samples. We performed normalization by dividing each variable by the value of the variable measured at the beginning of the experiment. We observe that, after 16 days from the beginning of the experiment, R, I_gs_, and I_0_ are reduced by 63%, 92%, and 37%, respectively. Moreover, R and I_gs_—the variables that show maximum reduction—exhibit a similar trend. For this configuration, (i) R has a significant reduction after 16 days; (ii) the PEDOT:PSS channel conductivity (∝I_0_) is minimally affected by the experimental set-up; and, conversely, (iii) the experimental set-up has a strong influence on I_gs_, showing a one-order-of-magnitude decrease. Furthermore, (iv) the decrease in the system’s response, R, is ascribed mostly to a reduction in conductivity at the gate–electrolyte interface.

**Figure 3:**
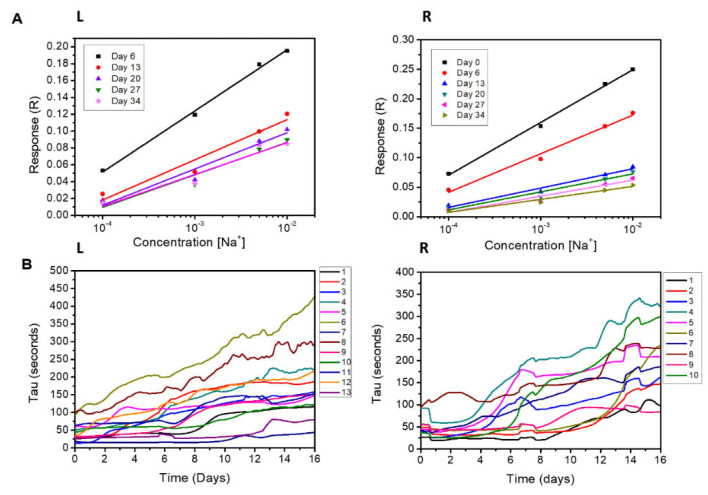
Average value of sensitivity (**A**) and tau variation over time (**B**) of the PEDOT:PSS channel with H_2_SO_4_ (10 devices, R) and EG (13 devices, L). The sensor sensitivity was measured with NaCl saline concentrations at 0.1 mM, 1 mM, 5 mM, and 10 mM for days 0, 6, 13, 20, 27, and 34 (**A**).

To further assess the performance of the devices over time, we determined the response (R) of all tested devices by measuring the solutions of sodium chloride with concentrations varying in the 0.1mM-10mM range at different time steps, i.e., at day 6, 13, 20, 27, and 34 from the beginning of the experiments (Figure 3A, L, Table S1a). On a log scale, the response is described by a simple linear regression. The results show the following: (i) the response (R) drops from 0.072 to 0.047 going from day 6 to day 13, with a variation of 35% in seven days, and (ii) from day 13 to day 34, the response shows a quasi-constant behavior, with a variation of 21% in 21 days, achieving a final value of 0.037. Thus, while the sensor device response decreases over time, this contraction is less relevant than other parameters of the devices reported in Figure 2: in the examined lifespan, the OECT is always sensitive to the salt in the solution for concentrations above 1 mM.

For the tested sensor devices’ outputs, we determined the value of the time constant *tau* (τ) to estimate the efficiency of de-doping at the channel [11,31]. Figure 3B, L shows that *tau* increases with time: the system’s inertia (τ) seems to grow with the number of days elapsed from the beginning of the experiment. The variation in the time constant *tau* as a function of the response R is reported in Figure S1a, showing an *inversely* proportional trend for at least up to day 16. The lower the values of *tau*, the faster the de-doping reaction at the channel. The results reported in Figure 3A,B show that the degradation of the sensor reduces the de-doping process and its speed.

### 3.2. Post-Treatment with Sulfuric Acid

We evaluated the performance of 10 sensors treated with sulfuric acid as explained in the Methods section. As in the previous case, I_gs_ decreases with time (Figure 2A, R). For 6 out of the 10 sensors, the current reduction begins between day 4 and 8. In contrast, four of the analyzed sensors maintain a constant value of current up to approximately day 8 (Figure 2A, R). As previously reported, I_0_ smoothly decreases with time (Figure 2B, R). The values of modulation (R) are comprised between 0.1 and 0.4 at the beginning of the experiment. After 16 days, R falls in the 0.0–0.25 range (Figure 2C, R). The degradation of the OECT sensor devices starts for six of the tested OECT devices beyond day 8, and for four of the tested devices, it starts between day 4 and day 8. For this configuration, the normalized average values of R, Igs, and I_0_ (Figure 2D, R) undergo an overall reduction of 69%, 93%, and 31%, respectively, after 16 days. Similar to the devices treated with ethylene glycol (EG), the channel current (I_0_) is minimally affected by the sulfuric acid treatment. The most appreciable variation is found for the gate–channel current (I_gs_).

We evaluated the sensitivity (R) of the sensor devices treated with sulfuric acid. Figure 3A, R and Table S1b show the modulation (R) of the sensor devices at different time steps from the beginning of the experiment, i.e., after 0, 6, 13, 20, 27, and 34 days, as measured for a solution of sodium chloride in deionized water with a concentration varying between 0.1 and 10 mM. At day 0, the average sensitivity is 0.089, and this decreases to 0.032 at day 13, showing a 64% decrease. Then, the sensitivity change becomes milder, with values of R moving from 0.032 at day 13 to 0.022 at day 34, with a reduction of 31% in 21 days. The change in sensitivity is more relevant in the first 14 days of the experiment.

The time constant (tau) associated with the measurements for all reported cases varies between 25 and 100 s at day 0 and increases to an interval in the range of 70–350 s at day 16 (Figure 3B, R). As previously reported, the time constant tau and the response R stand in an inverse relationship (Figure S1b): this suggests that the mechanism of degradation of the polymer is the same for both sample treatments.

### 3.3. Image Analysis of the PEDOT:PSS Polymer Fiber

To investigate the levels and the mechanism of degradation, we performed an image analysis of the PEDOT:PSS OECT fiber channel. We measured the RGB parameters of the PEDOT: PSS thread before and after the experiments and compared the results. The PEDOT:PSS thread, before use, is typically black (Figure S2: new wire) with a normalized grayscale value of ~0.113 as measured in correspondence to the channel (Figure S2a for test in Section 3.1) and a value of ~0.087 at the gate (Figure S2b for test in Section 3.2). After the tests, the PEDOT: PSS fiber with EG shows a grayscale value of 0.145 at the channel and a value of 0.269 at the gate, with an increment, with respect to the pristine fiber, of 22% and 58%, respectively (Figure S2a). Similarly, for the sulfuric acid treatment, the PEDOT: PSS fiber at the end of the measurements has a normalized grayscale value of ~0.104 at the channel and a value of 0.365 at the gate, with a brightness increment of 16% and the 76%, respectively (Figure S2b).

Moreover, the fiber at the gate appears to be frayed in both experimental conditions, suggesting a detachment of the PEDOT:PSS during the experimental tests for both treatments. This observation, along with the evidence that the I_gs_ variation with time is much higher than that of I_0_, suggests that PEDOT detachment at the gate can be the main cause of the device’s lifetime decrease. As a consequence, V_g_ is expected to be a critical parameter that can affect gate degradation.

**Figure 4:**
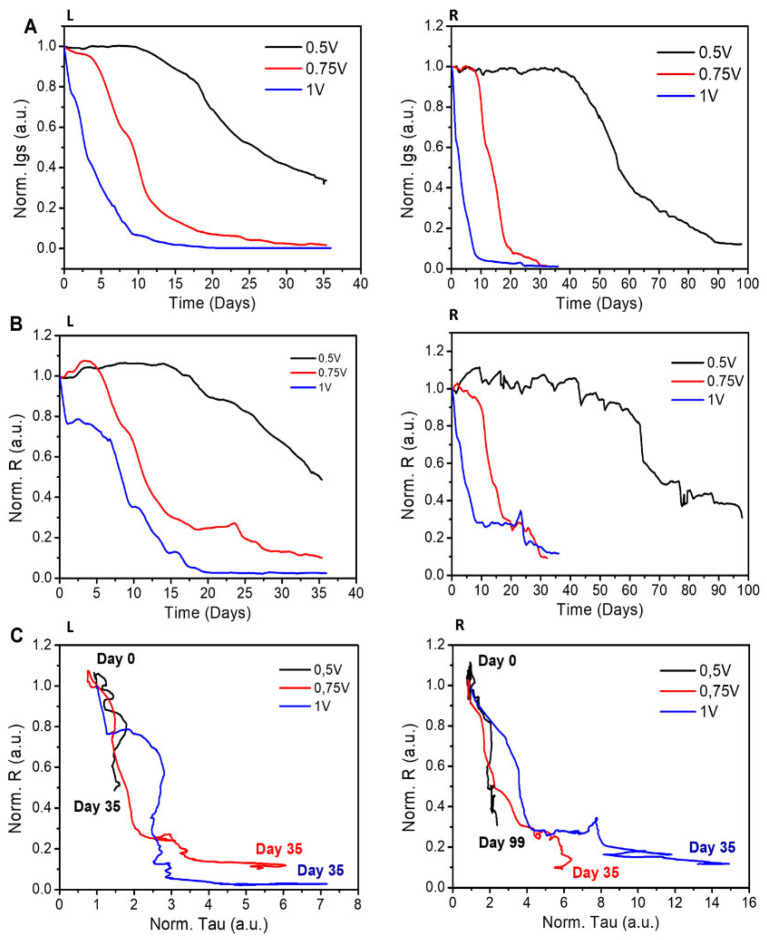
Normalized average of gate–source current (**A**) and response (**B**) at V_g_ = 1 V (blue line), 0.75 V (red line), and 0.5 V (black line). (**C**) shows the correlation between the normalized average R and tau over time. The average parameters over time are with respect to 3 sensors prepared with the EG method (L) for 35 days and the SA treatment (R) for 99 days.

We therefore performed additional experiments to study the effect of the gate voltage on the device’s lifetime. In these experiments, the voltage was varied in the 0.5–1 V range and periodically switched with a duty cycle of 6 min. The devices prepared using either (i) EG as a plasticizer or (ii) sulfuric acid in the post-treatment step were tested. For each combination of voltage and sample preparation, we repeated the experiments using three different samples. In Figure 4, we report the normalized values of R, I_gs_, and tau determined for each tested case. Then, we used a simple linear model to correlate the lifetime of the device to V_g_.

### 3.4. Effect of the Applied Voltage on Device Lifetime for EG Samples

In the case of ethylene glycol used as an additive in preparing the PEDOT:PSS solution for the device, both I_gs_ (Figure 4A, L) and R (Figure 4B, L) exhibit a more constant behavior as the values of gate voltage decrease. In Figure 4C, L, the reduction in R—from 1 to 0.025—is more relevant at 1 V and 0.75 V of gate voltage compared to the drop of R—from 1 to 0.49—experienced by the sensor device at 0.5 V. This difference is also reflected in *τ*, which, for higher values of V_g_, varies from 1 to the 3.5–7.5 interval, and, for lower values of V_g_, just reaches the 1.5 limit. This result suggests that the V_g_ potential affects the OECT response over time.

### 3.5. Effect of the Applied Voltage on Device Lifetime for SA Samples

In these experiments, the devices were tested for 100 days. In the case of the SA samples, we observe that both Igs (Figure 4A, R) and R (Figure 4B, R) show a longer lifetime when the gate voltage is reduced. In particular, for a gate voltage of 0.5 V, R is almost constant up to 60 days. These results indicate that (i) the duration of the devices increases if the values of gate voltage are reduced, and (ii) the SA samples show higher stability than the EG samples. The correlation between the normalized R and the normalized tau is shown in Figure 4C, R. There are three separate regions in the R–tau diagram: for R > 0.9 (i), for R falling in the 0.3–0.9 range (ii), and for R < 0.3 (iii). In these regions, respectively, (i) tau does not vary with time and all three tests have the same trend; (ii) R decreases and tau increases—the variation of tau is more relevant when a higher V_g_ is applied; and (iii) tau shows a very high sensitivity to time for V_g_ of 1 V and 0.75 V.

### 3.6. Correlation between the Lifetime of Device and V_g_

**Figure 5:**
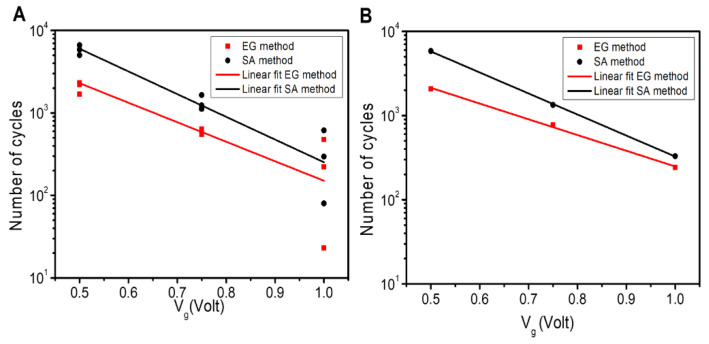
Number of on/off cycles at which I_gs_ reduces by 20% as a function of gate voltages for 3 devices (**A**), and the average of the 3 devices (**B**) for the EG method and with sulfuric acid (Acid) as the post-treatment.

To quantitatively evaluate duration and stability, we report in Figure 5A,B in a logarithmic scale the number of cycles before failure (lifetime)—for devices treated with either ethylene glycol or sulfuric acid—as a function of V_g_.

Sensor failure, i.e., a significant loss of functionality and performance, is defined as the condition for which the gate–source current (I_gs_) is reduced to 20% of its initial value. The diagrams indicate that the logarithm of the lifetime of the devices decreases linearly with V_g_ (Adj. R-Square > 0.95). Moreover, the data in the diagrams indicate that sample treatment with sulfuric acid is more effective than using ethylene glycol as a plasticizer. By fitting the data in Table 1 to a simple linear model of the form y=a+b x, where y is the logarithm of a device’s lifetime, x is the voltage expressed in volts, and a and b are the constants, we found that a=5.154 for sulfuric acid and a=4.542 for ethyl glycol, showing a 12% increment. For the slopes of the curves b, we found that b=2.752/Volt for sulfuric acid and b=2.364/Volt for ethylene glycol, showing a 14% increment. The simple linear model that we used to fit the data predicts that the device may work efficiently for more than 1000 cycles for a voltage value lower than 0.65 V and 0.78 V for ethylene glycol and sulfuric acid as the sample treatment, respectively.

## 4. Discussion

Textile OECTs based on the PEDOT: PSS conductive polymer enable an ideal interface for advanced biocompatible sensors that can find application in various fields, such as IoT technology, tissue engineering and regenerative medicine, precision medicine, and precision agriculture. Here, we examined how different sample treatments (i.e., using either ethylene glycol as a plasticizer or sulfuric acid as a post-treatment agent) and operation set-ups (i.e., different values of gate voltages) influence the lifetime of PEDOT:PSS organic electrochemical transistors.

We found that the deterioration over time is non-constant. After a phase when the device performance is stable, I_gs_, R, and the sensitivity of the OECT fall sharply, while at the same time, tau increases, showing an inverse trend when compared to R. Additionally, the drift of the device performance over time is similar for each type of fiber treatment tested. This mechanism depends, to a large extent, on the gate integrity, as examined by the RGB analysis, and its ability to pump cations into the channel. In consideration of these results, we modified the voltage of the gate from 1 V to 0.5 V. The results indicated that low V_g_ values contribute to enhancing the lifetime and reducing the degradation of the sensor devices. Notably, the plot of the lifetime of the devices as a function of the gate voltage has a linear behavior in the semi-logarithmic plot (lifetime is expressed as the number of cycles before failure). Finally, this experimental test campaign indicated that the devices treated with SA have a longer lifetime compared to the OECTs obtained using EG as the plasticizer. The better performance after the SA treatment can be correlated to the process of gate degradation. The SA and EG sample treatments are two of the many existing methods to increase PEDOT:PSS conductivity [20,21]. The EG treatment increases conductivity by increasing carrier mobility, carrier density, and interchain interaction without evidence of increasing crystallinity [20,32]. The SA immersion is one of the best ways to increase electrical properties, such as conductivity and mechanical and thermal resistance, due to increased PEDOT:PSS crystallinity and a decrease in PSS amount [20,33]. The results of this study suggest that high positive values of voltages—applied to the PEDOT:PSS in an aqueous solution—deteriorate the structure of the polymer over time. The increase in crystallinity could be a key factor to increase the lifetime of the sensor devices. Moreover, reducing the PSS amount in the SA treatment increases the hydrophobicity of the PEDOT:PSS thread, which can reduce gate degradation [33]. In addition, low V_g_ values enhance the lifetime and reduce the degradation of the sensor devices. This is particularly evident for V_g_ = 0.5 V. The plot of the lifetime of the devices as a function of the voltage at the gate has a linear behavior in the semi-logarithmic plot: this allows the prediction of the devices’ lifetime as a function of the experimental parameters.

## 5. Conclusions

We examined the long-term stability of devices obtained by two different treatments of PEDOT: PSS threads: using ethylene glycol as a plasticizer or using sulfuric acid in a post-treatment fabrication phase. In a preliminary test campaign, we analyzed the stability of the device using a voltage of 1 V at the gate. Then, we varied the value of V_g_ in the 0.5–1 V range. The results indicate that the principal source of malfunctioning of the devices is attributable to the erosion and detachment of the PEDOT:PSS polymer at the gate, which is caused by the applied voltage. A similar erosion leads to, as an effect, a reduction in the gate–source current (Igs) and in the number of cations that can de-dope the PEDOT: PSS in the channel. The devices treated with sulfuric acid, because of an increased level of crystallinity of PEDOT:PSS, show an increased lifetime. We showed that the lifetime of the sulfuric acid-treated samples can be enhanced even further by reducing the voltage at the gate from 1 V to 0.5 V. For these values of the model parameters, the PEDOT:PSS OECTs are stable over 80% for over 30 days (3600 measured cycles).

## Figures and Tables

**Table 1:** Linear fit parameters obtained from the graph in Figure 5.

	*y* = *a* + *b* ∗ *x*	Value	Standard Error	Adj. R-Square
Ethylene Glycol	a	4.542	0.260	0.961
	b	−2.364	0.335	
Sulfuric Acid	a	5.154	0.080	0.997
	b	−2.752	0.103	

## Data Availability

Data are available upon reasonable request to the corresponding Authors.

## Supplementary Materials

The following supporting information can be downloaded at: https://www.mdpi.com/article/10.3390/ma16051861/s1, Figure S1: average R-tau correlation up to 16 days (B) for 13 devices with EG method (B) and the SA treatment (A), as for Figure 2. Figure S2: Image analysis of the textile fiber functionalized with PEDOT:PSS: average RGB normalized grayscale value for channel and picture for functionalized fibers after 16 days of continuous measurements for gate, channel, and a functionalized fiber never used (New wire), for samples with: (A) addition of Ethylene glycol (B) Sulfuric Acid treatment. Table S1: values, standard errors and R2 of the linear fitting of the response in time showed in Figure 3A for devices with the PEDOT:PSS channel with H2SO4 (10 devices, A) and EG (13 devices, B).

## References

[1] Donahue M.J., Sanchez-Sanchez A., Inal S., Qu J., Owens R.M., Mecerreyes D., Malliaras G.G., Martin D.C. (2020). Tailoring PEDOT properties for applications in bioelectronics. Mater. Sci. Eng. R Rep..

[2] Tarabella G., Mahvash Mohammadi F., Coppedè N., Barbero F., Iannotta S., Santato C., Cicoira F. (2013). New opportunities for organic electronics and bioelectronics: Ions in action. Chem. Sci..

[3] Lee I., Lee K. (2015). The Internet of Things (IoT): Applications, investments, and challenges for enterprises. Bus. Horiz..

[4] Madakam S., Ramaswamy R., Tripathi S. (2015). Internet of Things (IoT): A Literature Review. J. Comput. Commun..

[5] Rogers B., Zonfrilli C., Kreher T., Della R.T. (2017). The Internet of Things: From theory to reality. Forbes Insight.

[6] Volkov A.V., Wijeratne K., Mitraka E., Ail U., Zhao D., Tybrandt K., Andreasen J.W., Berggren M., Crispin X., Zozoulenko I.V. (2017). Understanding the Capacitance of PEDOT:PSS. Adv. Funct. Mater..

[7] Gentile F., Coppedè N., Tarabella G., Villani M., Calestani D., Candeloro P., Iannotta S., Di Fabrizio E. (2014). Microtexturing of the conductive PEDOT:PSS Polymer for superhydrophobic organic electrochemical transistors. BioMed Res. Int..

[8] Verma D., Singh K.R., Yadav A.K., Nayak V., Singh J., Solanki P.R., Singh R.P. (2022). Internet of things (IoT) in nano-integrated wearable biosensor devices for healthcare applications. Biosens. Bioelectron. X.

[9] Strakosas X., Bongo M., Owens R.M. (2015). The organic electrochemical transistor for biological applications. J. Appl. Polym. Sci..

[10] Battista E., Lettera V., Villani M., Calestani D., Gentile F., Antonio P., Iannotta S., Zappettini A., Netti P.A., Iannotta S. (2017). Enzymatic sensing with laccase-functionalized textile organic biosensors. Org. Electron..

[11] Coppedè N., Tarabella G., Villani M., Calestani D., Iannotta S., Zappettini A. (2014). Human stress monitoring through an organic cotton-fiber biosensor. J. Mater. Chem. B.

[12] Vurro F., Janni M., Coppedè N., Gentile F., Manfredi R., Bettelli M., Zappettini A. (2019). Development of an in vivo sensor to monitor the effects of vapour pressure deficit (VPD) changes to improve water productivity in agriculture. Sensors.

[13] Tarabella G., Villani M., Calestani D., Mosca R., Iannotta S., Zappettini A., Coppedè N. (2012). A single cotton fiber organic electrochemical transistor for liquid electrolyte saline sensing. J. Mater. Chem..

[14] Coppedè N., Giannetto M., Villani M., Lucchini V., Battista E., Careri M., Zappettini A. (2020). Ion selective textile organic electrochemical transistor for wearable sweat monitoring. Org. Electron..

[15] Coppedè N., Janni M., Bettelli M., Maida C.L., Gentile F., Villani M., Ruotolo R., Iannotta S., Marmiroli N., Marmiroli M. (2017). An in vivo biosensing, biomimetic electrochemical transistor with applications in plant science and precision farming. Sci. Rep..

[16] Bandodkar A.J., Wang J. (2014). Non-invasive wearable electrochemical sensors: A review. Trends Biotechnol..

[17] Koh A., Kang D., Xue Y., Lee S., Pielak R.M., Kim J., Hwang T., Min S., Banks A., Bastien P. (2016). A soft, wearable microfluidic device for the capture, storage, and colorimetric sensing of sweat. Sci. Transl. Med..

[18] Amato D., Montanaro G., Vurro F., Coppedé N., Briglia N., Petrozza A., Janni M., Zappettini A., Cellini F., Nuzzo V. (2021). Towards In Vivo Monitoring of Ions Accumulation in Trees: Response of an in Planta Organic Electrochemical Transistor Based Sensor to Water Flux Density, Light, Vapor Pressure Deficit Variation. Appl. Sci..

[19] Janni M., Coppede N., Bettelli M., Briglia N., Petrozza A., Summerer S., Vurro F., Danzi D., Cellini F., Marmiroli N. (2019). In Vivo Phenotyping for the Early Detection of Drought Stress in Tomato. Plant Phenomics.

[20] Shi H., Liu C., Jiang Q., Xu J. (2015). Effective Approaches to Improve the Electrical Conductivity of PEDOT:PSS: A Review. Adv. Electron. Mater..

[21] Fan X., Nie W., Tsai H., Wang N., Huang H., Cheng Y., Wen R., Ma L., Yan F., Xia Y. (2019). PEDOT:PSS for Flexible and Stretchable Electronics: Modifications, Strategies, and Applications. Adv. Sci..

[22] Kayser L.V., Lipomi D.J. (2019). Stretchable Conductive Polymers and Composites Based on PEDOT and PEDOT:PSS. Adv. Mater..

[23] Al-Oqla F.M., Sapuan S.M., Anwer T., Jawaid M., Hoque M.E. (2015). Natural fiber reinforced conductive polymer composites as functional materials: A review. Synth. Met..

[24] Zeglio E., Inganäs O. (2018). Active Materials for Organic Electrochemical Transistors. Adv. Mater..

[25] D’Angelo P., Marasso S.L., Verna A., Ballesio A., Parmeggiani M., Sanginario A., Tarabella G., Demarchi D., Pirri C.F., Cocuzza M. (2019). Scaling Organic Electrochemical Transistors Down to Nanosized Channels. Small.

[26] ElMahmoudy M., Inal S., Charrier A., Uguz I., Malliaras G.G., Sanaur S. (2017). Tailoring the Electrochemical and Mechanical Properties of PEDOT:PSS Films for Bioelectronics. Macromol. Mater. Eng..

[27] Coppede N., Vurro F., Manfredi R., Janni M., Zappettini A., Gentile F. (2019). Introducing State Variables in Organic Electrochemical Transistors With Application to Biophysical Systems. IEEE Sens. J..

[28] Malara N., Gentile F., Coppedè N., Coluccio M.L., Candeloro P., Perozziello G., Ferrara L., Giannetto M., Careri M., Castellini A. (2018). Superhydrophobic lab-on-chip measures secretome protonation state and provides a personalized risk assessment of sporadic tumour. npj Precis. Oncol..

[29] Güne A., Kalkan H., Efkan K. (2016). Optimizing the Color-to-Grayscale Conversion for Image Classification.

[30] Prats-Montalbán J.M., de Juan A., Ferrer A. (2011). Multivariate image analysis: A review with applications. Chemom. Intell. Lab. Syst..

[31] Coppede N., Villani M., Gentile F. (2014). Diffusion Driven Selectivity in Organic Electrochemical Transistors. Sci. Rep..

[32] Ouyang J., Xu Q., Chu C.W., Yang Y., Li G., Shinar J. (2004). On the mechanism of conductivity enhancement in poly(3,4- ethylenedioxythiophene):poly(styrene sulfonate) film through solvent treatment. Polymer (Guildf).

[33] Kim S.-M., Kim C.-H., Kim Y., Kim N., Lee W.-J., Lee E.-H., Kim D., Park S., Lee K., Rivnay J. (2018). Influence of PEDOT:PSS crystallinity and composition on electrochemical transistor performance and long-term stability. Nat. Commun..

