# Supplementary material for: Long-Term Stability in Electronic Properties of Textile Organic Electrochemical Transistors for Integrated Applications
Source: Materials (Basel). 2023 Feb 24;16(5):1861. doi: 10.3390/ma16051861 (PMC10003982; doi:10.3390/ma16051861)
Supplement: Supplementary file 1 [file materials-16-01861-s001.zip › materials-2142236-supplementary.pdf]

A	y = a + b*x	Value	Standard Error	Adj. R-Square
Day 6	a	0.342	0.009	0.994
	b	0.073	0.003	
Day 13	a	0.209	0.023	0.920
	b	0.048	0.008	
Day 20	a	0.184	0.020	0.927
	b	0.043	0.007	
Day 27	a	0.164	0.018	0.927
	b	0.039	0.006	
Day 34	a	0.162	0.019	0.918
	b	0.038	0.006	

B	y = a + b*x	Value	Standard Error	Adj. R-Square
day 0	a	0.428	0.010	0.996
	b	0.089	0.003	
day 6	a	0.303	0.014	0.983
	b	0.066	0.005	
day 13	a	0.147	0.010	0.968
	b	0.033	0.003	
day 20	a	0.134	0.014	0.931
	b	0.031	0.005	
day 27	a	0.117	0.014	0.918
	b	0.027	0.005	
day 34	a	0.095	0.008	0.956
	b	0.022	0.003	

Table S1. 1: Table with values, standard error and R^2 of response linear fitting in time showed in Figure 3A for devices with the PEDOT:PSS channel with H_2SO_4 (10 devices, A)) and EG (13 devices, B).

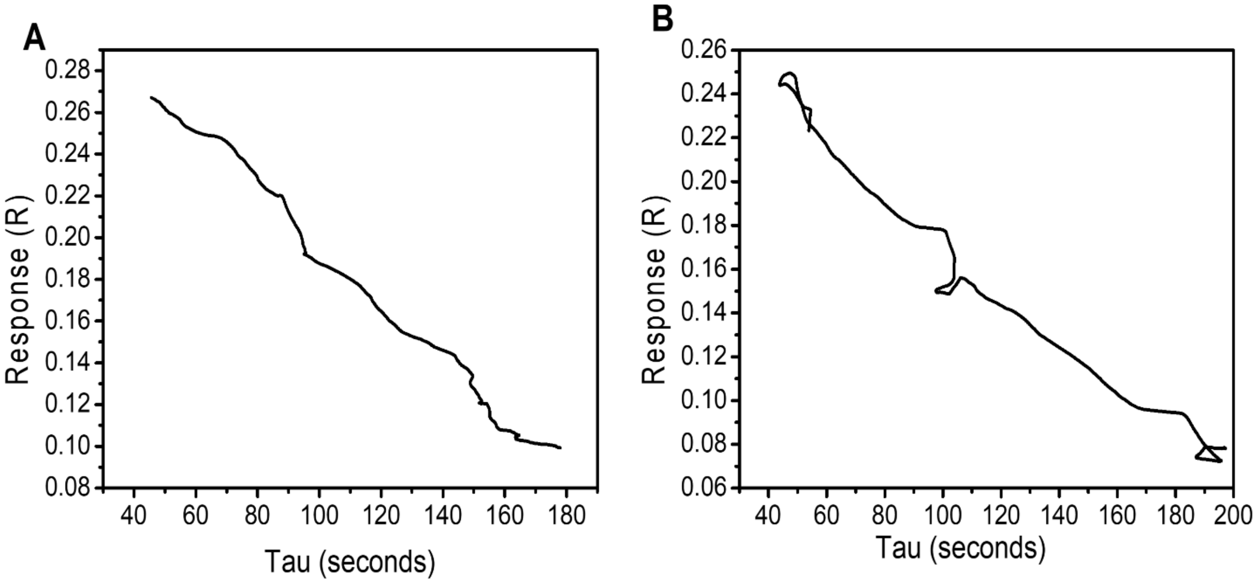

Figure S1.1: and the average R-tau correlation up to 16 days (B) for 13 devices with EG method (dx) and the SA treatment (sx), as for Figure 2.

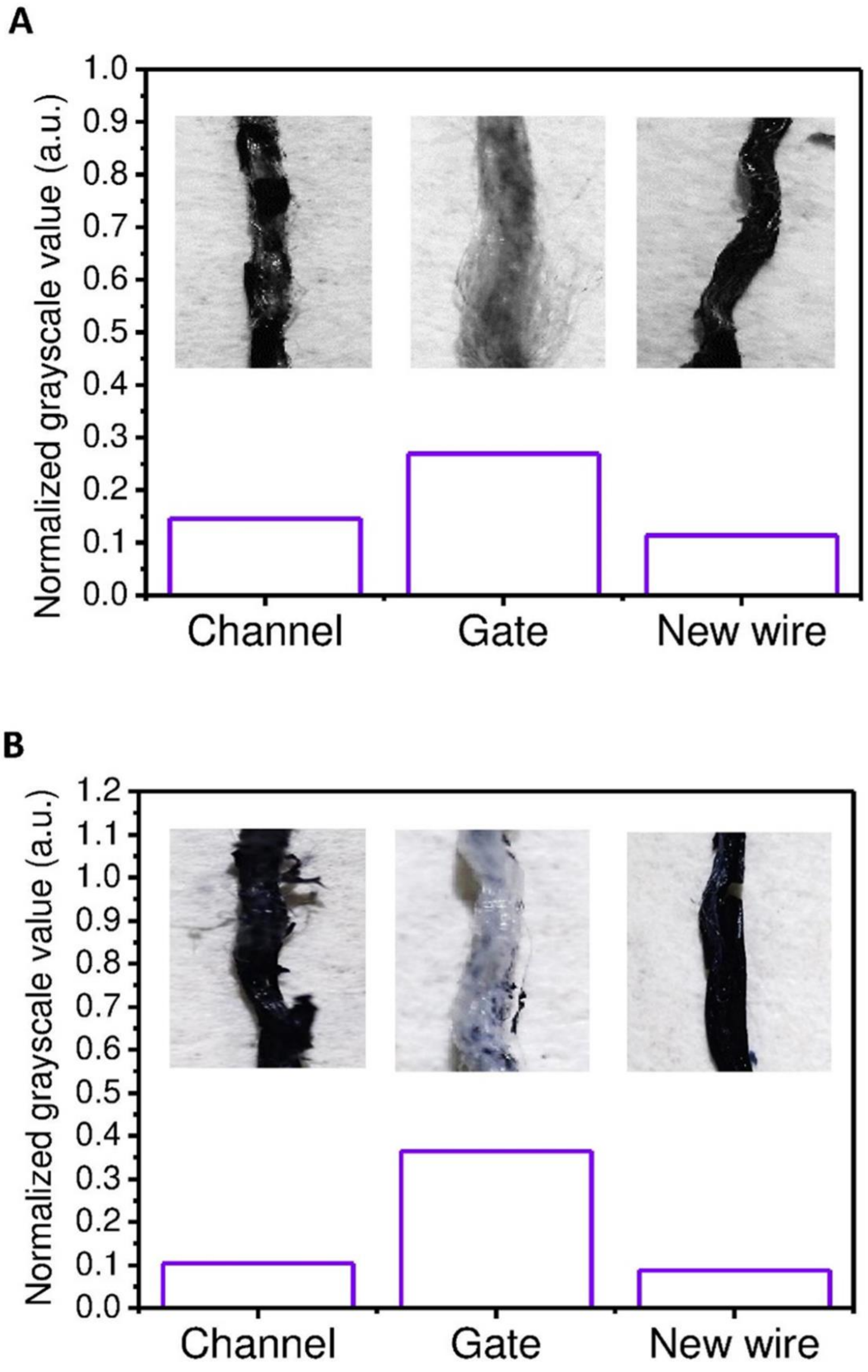

Figure S2. 2: Image analysis of the textile fiber functionalized with PEDOT:PSS: average RGB normalized grayscale value for channel and picture for functionalized fibers after 16 days of continuous measurements for gate, channel, and a functionalized fiber never used (New wire), for samples with: A) addition of Ethylene glycol B) Sulfuric Acid treatment.
